# Use of the Shizuoka Hip Fracture Prognostic Score (SHiPS) to Predict Long‐Term Mortality in Patients With Hip Fracture in Japan: A Cohort Study Using the Shizuoka Kokuho Database

**DOI:** 10.1002/jbm4.10743

**Published:** 2023-04-05

**Authors:** Emi Ohata, Eiji Nakatani, Hideaki Kaneda, Yoh Fujimoto, Kiyoshi Tanaka, Akira Takagi

**Affiliations:** ^1^ Graduate School of Public Health Shizuoka Graduate University of Public Health Shizuoka Japan; ^2^ 4DIN Ltd Tokyo Japan; ^3^ Translational Research Center for Medical Innovation, Foundation for Biomedical Research and Innovation at Kobe Kobe Japan; ^4^ Department of Pediatric Orthopedics Shizuoka Children's Hospital Shizuoka Japan; ^5^ Department of General Internal Medicine Shizuoka General Hospital Shizuoka Japan; ^6^ Faculty of Nutrition Kobe Gakuin University Kobe Japan; ^7^ Department of Otolaryngology Shizuoka General Hospital Shizuoka Japan

**Keywords:** HIP FRACTURE, LONG‐TERM MORTALITY, POPULATION‐BASED STUDIES, PREOPERATIVE SCORING SYSTEM, PROGNOSIS

## Abstract

Hip fractures are common in patients of advanced age and are associated with excess mortality. Rapid and accurate prediction of the prognosis using information that can be easily obtained before surgery would be advantageous to clinical management. We performed a population‐based retrospective cohort study using an 8.5‐year Japanese claims database (April 2012–September 2020) to develop and validate a predictive model for long‐term mortality after hip fracture. The study included 43,529 patients (34,499 [79.3%] women) aged ≥65 years with first‐onset hip fracture. During the observation period, 43% of the patients died. Cox regression analysis identified the following prognostic predictors: sex, age, fracture site, nursing care certification, and several comorbidities (any malignancy, renal disease, congestive heart failure, chronic pulmonary disease, liver disease, metastatic solid tumor, and deficiency anemia). We then developed a scoring system called the Shizuoka Hip Fracture Prognostic Score (SHiPS); this system was established by scoring based on each hazard ratio and classifying the degree of mortality risk into four categories based on decision tree analysis. The area under the receiver operating characteristic (ROC) curve (AUC) (95% confidence interval [CI]) of 1‐year, 3‐year, and 5‐year mortality based on the SHiPS was 0.718 (95% CI, 0.706–0.729), 0.736 (95% CI, 0.728–0.745), and 0.758 (95% CI, 0.747–0.769), respectively, indicating good predictive performance of the SHiPS for as long as 5 years after fracture onset. Even when the SHiPS was individually applied to patients with or without surgery after fracture, the prediction performance by the AUC was >0.7. These results indicate that the SHiPS can predict long‐term mortality using preoperative information regardless of whether surgery is performed after hip fracture.

## Graphical abstract

A retrospective cohort study was used to identify variables predicting mortality in those suffering first hip fracture, with sex, fracture site, nursing care certification, and diseases including malignancy and heart failure being identified. These variables were used to construct a points‐based scoring system called Shizuoka Hip Fracture Prognostic Score (SHiPS), which successfully predicted mortality up to 5 years after fracture.

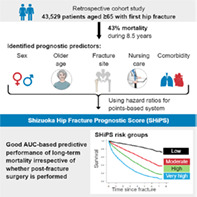

© 2023 The Authors. *JBMR Plus* published by Wiley Periodicals LLC on behalf of American Society for Bone and Mineral Research.

## Introduction

The high incidence of hip fractures in the advanced‐age population is a common public health problem. With the growth and aging of the population, the number of hip fractures worldwide is expected to increase from 1.26 million in 1990 to 4.50 million by 2050.^(^ ^1^, ^2^ ^)^ This rate will accelerate, particularly in Asia, where the population is aging more rapidly. In Japan, where the aging population is the largest in the world, the number of hip fractures was 193,400 in 2017^(^ ^3^ ^)^ and is estimated to be 320,000 by 2040.^(^ ^4^ ^)^ Hip fractures in patients of advanced age often result in loss of independent mobility and higher mortality than other diseases.^(^ ^5^, ^6^ ^)^ Understanding the prognostic factors for hip fractures and identifying high‐risk patients are essential for providing appropriate medical care and management and efficiently distributing healthcare resources.

Previous studies have revealed various prognostic factors after hip fractures, such as sex, age, body mass index (BMI), comorbidities, prescribed medications, fracture type, prefracture residence, and prefracture mobility.^(^ ^7^, ^8^, ^9^, ^10^, ^11^, ^12^, ^13^, ^14^, ^15^, ^16^, ^17^, ^18^, ^19^, ^20^, ^21^, ^22^, ^23^ ^)^ In addition, scoring systems such as the Charlson comorbidity index (CCI), American Society of Anesthesiologists grade, Nottingham Hip Fracture Score (NHFS), and Physiological and Operative Severity Score for enUmeration of Mortality and Morbidity (POSSUM), as well as some machine learning approaches have been created and validated to predict mortality after hip fracture.^(^ ^24^, ^25^, ^26^, ^27^, ^28^, ^29^, ^30^, ^31^ ^)^ However, the results of these studies have several limitations when applied to actual clinical practice. The predictive mortality in these studies was relatively short‐term (eg, 30 days or 1 year), and the applicability of the results to Asian patients is unclear from an ethnic perspective.

Exploration of prognostic factors after hip fracture in Japan, a leading Asian country for aging, may contribute to appropriate medical management and reduced post‐onset mortality. In this study, we used the Japanese insurance claims database for 8.5 years from 2012 to 2020 to identify preoperative prognostic factors for development of a scoring system, the Shizuoka Hip Fracture Prognostic Score (SHiPS), to predict long‐term (5‐year) mortality after hip fracture.

## Subjects and Methods

### Data sources

The Shizuoka Kokuho Database (SKDB) is an insurance claims database in Shizuoka prefecture, Japan, that was expanded to 2,398,393 individuals (1,303,667 [54.4%] women) collected over the 8.5‐year period from April 2012 to September 2020.^(^ ^32^ ^)^ Shizuoka is located in central Japan, has a population of approximately 3.7 million people, and is characterized by the standard climate, demographics, and economy of Japan. The SKDB includes the data from the National Health Insurance (NHI) for individuals aged <75 years and the Latter‐stage Elderly Medical Care System (LSEMCS) for individuals aged ≥75 years among residents in Shizuoka prefecture. These data comprise information on age; sex; diagnosis based on the International Statistical Classification of Diseases and Related Health Problems, Tenth Revision (ICD‐10); medical treatment; prescribed medications and their dates of administration; the level of Japanese nursing care certification; and the accurate date of death. The SKDB has been used as a data source in several published studies.^(^ ^33^, ^34^, ^35^ ^)^

### Study design and population

This population‐based retrospective cohort study was performed using the SKDB. The cohort was defined as the period from the registration date of health insurance agencies or April 1, 2012, whichever occurred later, to the date of insurance withdrawal (for transferring to other insurance, or to welfare public assistance, or death) or September 30, 2020, whichever occurred earlier. The index date was determined as the first onset of hip fracture in individuals with at least 1 year of continuous subscribership after cohort entry. Patients with hip fractures were defined by ICD‐10 code S72.0 (femoral neck), S72.1 (pertrochanteric), and S72.2 (subtrochanteric).

The baseline period was set 1 year before the onset of hip fracture and was used to exclude patients aged <65 years or who had already experienced any hip fracture during that time. In addition, patients with two or more ICD‐10 codes for this fracture site were excluded from the study population because the exact fracture site could not be determined based on the receipt data.

### Outcome and covariates

The primary outcome was death after the first onset of hip fracture, and the duration from the onset of hip fracture to death was observed. The baseline period was used to collect the patients' demographic information and comorbidities. We used the CCI and Elixhauser comorbidity index (ECI), both of which are widely used for comorbidities, as potential predictive factors.^(^ ^36^, ^37^ ^)^

We also collected the Japanese nursing care certification records, which are certified strictly based on a patient's physical and mental status, and could be an indicator of activities of daily living (ADL). The nursing care assessment, based on the process in Japan's long‐term care insurance system,^(^ ^38^ ^)^ is limited to persons aged ≥65 years or persons aged ≥40 years with specific diseases. The level of nursing care is categorized as requiring help level 1 or 2 and long‐term care level 1 to 5, and all persons who had been certified at any level were included as nursing care‐certified patients in this study.

### Timing of surgical approaches

We investigated whether surgical or conservative approaches were implemented after hip fracture. The surgical procedures included total hip arthroplasty, bipolar hip arthroplasty, and open surgery. Because the SKDB only provides information for the month in which the surgery was performed, the preoperative waiting times were classified as follows: within 1 month, 1 to 2 months, 2 to 3 months, 3 to 4 months, 4 to 5 months, 5 to 6 months, and more than 6 months after fracture onset.

### Statistical analysis

Continuous and categorical variables are summarized using mean ± standard deviation and frequency (percentage). To compare baseline characteristics between the patients who survived and those who died after hip fracture, the *t* test and chi‐squared test were used for continuous and categorical variables, respectively. The survival rate was calculated using the Kaplan‐Meier method, and the log‐rank test was used to compare groups.

To develop a mortality prediction scoring system after hip fracture, two‐thirds of all patients were randomly selected as the training data set. The remaining one‐third of patients were used as the test data set. Univariate and multivariate Cox proportional hazards regression analyses were conducted to explore prognostic factors using the training data set. We calculated the hazard ratio (HR), 95% confidence interval (CI) based on the Wald test, and corresponding *p* value. The variables used in the multivariate model were sex, age, season of onset, fracture site, nursing care certification, and CCI/ECI. Spearman's rank correlation coefficient was used to check correlations between potential predictors (correlated: ≥0.4). All potential independent predictors were entered into the multivariate model.

Next, HR values were converted to logarithms, multiplied by the same number, and then rounded to the nearest integer to develop the scores for ShiPS.^(^ ^39^ ^)^ The risk classification of the SHiPS was determined based on a conditional inference tree analysis. First, the data were sequentially divided into two groups according to the SHiPS. Next, the permutation test was used to compare the two groups, and the variable with the minimum *p* value was selected as the grouping node. This method was repeated for each subgroup until all separations were not without significance or the minimum node was reached. To evaluate the performance of the scoring system and the classification, Uno's C‐index was calculated throughout the entire survival time.^(^ ^40^ ^)^ To assess the predictive performance at the 1‐year, 3‐year, and 5‐year time points for scoring, time‐dependent areas under the receiver operating characteristic (ROC) curve (AUCs) were calculated.^(^ ^41^ ^)^

Because there are no missing values for all variables in this study, missing values were not imputed in all analyses. A two‐sided *p* value of <0.001 was considered statistically significant. All statistical analyses were carried out using SAS version 9.4 (SAS Institute Inc., Cary, NC, USA), R version 4.1.1 (The R Foundation for Statistical Computing, Vienna, Austria), and EZR version 1.54 (Saitama Medical Center, Jichi Medical University, Saitama, Japan), which is a graphical user interface for R.^(^ ^42^ ^)^

### Ethics statement

All patient‐related data were anonymized to protect the participants' confidentiality. The ethics committee of the Shizuoka Graduate University of Public Health approved the study protocol (SGUPH_2021_001_020).

## Results

### Study population and surgical treatment of hip fracture

A flowchart of this study is shown in Fig. S1. Among the 2,398,393 individuals (1,303,667 [54.4%] women) in the SKDB, 488,678 had <1 year of subscribership after cohort entry. Among the remaining individuals, 1,865,021 were excluded, among whom 1,851,115 had never experienced hip fracture and 13,906 had already had hip fracture during the baseline period. Therefore, 44,694 patients (35,116 [78.6%] women) were identified as having first‐onset hip fracture during our study period in all age groups. We then further excluded 1,165 of these patients (617 [53.0%] women) aged <65 years. Thus, 43,529 patients (34,499 [79.3%] women) were evaluated.

Of these patients, 32,025 (25,608 [80.0%] women) had undergone surgery after fracture onset and 11,504 (8,891 [77.3%] women) did not undergo surgery (also see Table 2).

### Characteristics of patients with hip fracture

To develop and validate a scoring system, the evaluated patients were randomly divided into training and test data sets. The training data set included 29,019 patients (23,012 [79.3%] women), and the test data set included 14,510 patients (11,397 [78.5%] women) (Fig. S1). The characteristics of the patients with first‐onset hip fracture in the training and test data sets are shown in Table 1. There were no significant differences in the patients' characteristics between the training and test data sets.

**Table 1:** Characteristics of Patients Who Sustained First Hip Fractures in Training and Test Data Sets

Variable	Category	Training data set (*n* = 29,019)			Test data set (*n* = 14,510)		
		Survival number (%)	Death number (%)	*p*	Survival number (%)	Death number (%)	*p*
*N* (%)		16,478 (56.8)	12,541 (43.2)		8,318 (57.3)	6,192 (42.7)	
Sex, *n* (%)	Male	2,627 (15.9)	3,290 (26.2)	<0.001	1,367 (16.4)	1,746 (28.2)	<0.001
	Female	13,851 (84.1)	9,251 (73.8)		6,951 (83.6)	4,446 (71.8)	
Age (years), mean ± SD		83.5 ± 7.3	87.3 ± 6.7	<0.001	83.7 ± 7.3	87.2 ± 6.7	<0.001
Age by group, *n* (%)	65 to <75 years	2,100 (12.7)	519 (4.1)	<0.001	994 (11.9)	241 (3.9)	<0.001
	75 to <85 years	6,502 (39.5)	3,363 (26.8)		3,296 (39.6)	1,700 (27.5)	
	85 to <95 years	7,040 (42.7)	6,989 (55.7)		3,565 (42.9)	3,468 (56.0)	
	≥95 years	836 (5.1)	1,670 (13.3)		463 (5.6)	783 (12.6)	
Season of onset, *n* (%)	January‐March	4,417 (26.8)	3,337 (26.6)	<0.001	2,223 (26.7)	1,677 (27.1)	<0.001
	April‐June	3,944 (23.9)	3,006 24.0)		1,965 (23.6)	1,431 (23.1)	
	July‐September	3,965 (24.1)	2,769 (22.1)		2,046 (24.6)	1,356 (21.9)	
	October‐December	4,152 (25.2)	3,429 (27.3)		2,084 (25.1)	1,728 (27.9)	
Fracture site, *n* (%)^a^							
S72.0	Presence	8,536 (51.8)	5,995 (47.8)	<0.001	4,359 (52.4)	3,022 (48.8)	<0.001
S72.1	Presence	5,614 (34.1)	4,527 (36.1)	<0.001	2,705 (32.5)	2,198 (35.5)	<0.001
S72.2	Presence	179 (1.1)	147 (1.2)	0.528	84 (1.0)	55 (0.9)	0.511
Nursing care certification, *n* (%)	Presence	7,649 (46.4)	8,999 (71.8)	<0.001	3,859 (46.4)	4,449 (71.9)	<0.001
Comorbidity, *n* (%)							
Cerebrovascular disease	Presence	5,148 (31.2)	4,802 (38.3)	<0.001	2,713 (32.6)	2,352 (38.0)	<0.001
Any malignancy	Presence	1,744 (10.6)	1,998 (15.9)	<0.001	919 (11.0)	1,033 (16.7)	<0.001
Dementia	Presence	4,604 (27.9)	5,000 (39.9)	<0.001	2,316 (27.8)	2,477 (40.0)	<0.001
AIDS/HIV	Presence	4 (0.0)	3 (0.0)	1	3 (0.0)	3 (0.0)	0.705
Myocardial infarction	Presence	552 (3.3)	666 (5.3)	<0.001	287 (3.5)	351 (5.7)	<0.001
Renal disease	Presence	1,270 (7.7)	1,674 (13.3)	<0.001	695 (8.4)	832 (13.4)	<0.001
Congestive heart failure	Presence	4,862 (29.5)	5,416 (43.2)	<0.001	2,523 (30.3)	2,662 (43.0)	<0.001
Peripheral vascular disease	Presence	2,378 (14.4)	2,020 (16.1)	<0.001	1,211 (14.6)	1,009 (16.3)	0.004
Chronic pulmonary disease	Presence	3,791 (23.0)	3,452 (27.5)	<0.001	1,971 (23.7)	1,779 (28.7)	<0.001
Rheumatic disease	Presence	896 (5.4)	602 (4.8)	0.016	419 (5.0)	314 (5.1)	0.939
Peptic ulcer disease	Presence	3,835 (23.3)	3,236 (25.8)	<0.001	1,972 (23.7)	1,664 (26.9)	<0.001
Mild liver disease	Presence	2,577 (15.6)	2,011 (16.0)	0.363	1,326 (15.9)	954 (15.4)	0.394
Diabetes without chronic complication	Presence	1,732 (10.5)	1,272 (10.1)	0.312	888 (10.7)	614 (9.9)	0.144
Diabetes with chronic complication	Presence	1,250 (7.6)	930 (7.4)	0.59	664 (8.0)	444 (7.2)	0.072
Hemiplegia or paraplegia	Presence	445 (2.7)	337 (2.7)	0.971	223 (2.7)	185 (3.0)	0.286
Moderate or severe liver disease	Presence	69 (0.4)	116 (0.9)	<0.001	28 (0.3)	74 (1.2)	<0.001
Metastatic solid tumor	Presence	208 (1.3)	408 (3.3)	<0.001	110 (1.3)	229 (3.7)	<0.001
Blood loss anemia	Presence	546 (3.3)	468 (3.7)	0.057	267 (3.2)	233 (3.8)	0.073
Deficiency anemia	Presence	3,254 (19.7)	3,357 (26.8)	<0.001	1,645 (19.8)	1,632 (26.4)	<0.001

Abbreviation: AIDS/HIV = acquired immune deficiency syndrome/human immunodeficiency virus; SD = standard deviation.

^a^ ICD‐10 codes were only considered if they were recorded alone. S72.0 Fracture of femoral neck, S72.1 Pertrochanteric fracture, S72.2 Subtrochanteric fracture.

Of the total patients, 43.2% in the training data set (median follow‐up time: 2.07 years) and 42.7% in the test data set (median: 2.09 years) died after sustaining the hip fracture. Compared with the patients who survived after the onset, those who died tended to be male, be older, be nursing care–certified, and have more comorbidities. The season of onset tended to be less in summer and more in winter. The most common fracture sites in patients who died were femoral neck fractures (S72.0) and pertrochanteric fractures (S72.1).

### Association between surgery after hip fracture and mortality

Table 2 shows patients who underwent surgery after a first hip fracture and those who did not, classified by survival and death. A total of 53.0% (*n* = 6,097) of patients without surgery and 39.5% (*n* = 12,636) with surgery died (*p* < 0.001). Almost all of the patients who underwent surgery, whether alive or dead, had surgery within 1 month of fracture. Table S1 also shows the characteristics of patients who underwent surgery after fracture onset and those who did not.

**Table 2:** Surgery After First‐Onset Hip Fracture Classified by Survival or Death

Treatment (*n* = 43,529)	Survival number (%) (*n* = 24,796)	Death number (%) (*n* =18,733)	*p*
Without surgery (*n* = 11,504)	5,407	6,097	<0.001
With surgery (*n* = 32,025)	19,389	12,636	
Within 1 month	18,902 (97.5)	12,372 (97.9)	
1 to <2 months	58 (0.3)	53 (0.4)	
2 to <3 months	24 (0.1)	19 (0.2)	
3 to <4 months	19 (0.1)	16 (0.1)	
4 to <5 months	23 (0.1)	11 (0.7)	
5 to <6 months	363 (1.9)	165 (1.3)	
6+ months	346 (1.8)	156 (1.2)	

### Identification of prognostic factors after hip fracture

We evaluated potential prognostic factors using univariate and multivariate Cox regression analyses with the training data set (*n* = 29,019) (Table 3). Of the variables with a *p* value of <0.001 in the univariate analysis, nursing care certification and dementia were correlated as judged by Spearman's rank correlation coefficient (absolute value of ≥0.4) (Table S2). We selected a nursing care–certified status in this study because it is more widely used in Japan than a dementia diagnosis, which could reflect a population with declining ADL due to various factors.

**Table 3:** Results of Univariate and Multivariate Cox Regression Analyses for Mortality in Training Data Set

Variable (reference)	Category	Training data set (*n* = 29,019)					
		Univariable model			Multivariable model		
		HR	95% CI	*p*	HR	95% CI	*p*
Sex (Female)	Male	1.85	1.78–1.93	<0.001	2.09	2.01–2.18	<0.001
Age (65 to <75 years)	75 to <85 years	1.73	1.58–1.90	<0.001	1.57	1.43–1.72	<0.001
	85 to <95 years	3.18	2.90–3.47	<0.001	2.65	2.41–2.90	<0.001
	≥95 years	5.67	5.14–6.26	<0.001	4.56	4.12–5.05	<0.001
Season of onset (January‐March)	April‐June	0.99	0.94–1.04	0.745			
	July‐September	0.98	0.93–1.03	0.392			
	October‐December	1.01	0.96–1.06	0.707			
Fracture site^a^ (Absence)							
S72.0	Presence	0.89	0.89–0.92	<0.001	1.13	1.07–1.18	<0.001
S72.1	Presence	1.20	1.15–1.24	<0.001	1.14	1.08–1.20	<0.001
S72.2	Presence	1.11	0.94–1.30	0.219			
Nursing care certification (Absence)	Presence	2.48	2.38–2.58	<0.001	2.11	2.02–2.19	<0.001
Comorbidity (Absence)							
Cerebrovascular disease	Presence	1.26	1.21–1.31	<0.001	1.04	1.01–1.08	0.026
Any malignancy	Presence	1.59	1.52–1.67	<0.001	1.29	1.22–1.36	<0.001
Dementia	Presence	1.67	1.61–1.73	<0.001	NA	NA	NA
AIDS/HIV	Presence	1.45	0.47–4.51	0.518			
Myocardial infarction	Presence	1.56	1.44–1.68	<0.001	1.13	1.04–1.22	0.003
Renal disease	Presence	1.82	1.73–1.92	<0.001	1.42	1.35–1.50	<0.001
Congestive heart failure	Presence	1.73	1.67–1.79	<0.001	1.32	1.27–1.37	<0.001
Peripheral vascular disease	Presence	1.11	1.06–1.17	<0.001	0.96	0.92–1.01	0.129
Chronic pulmonary disease	Presence	1.27	1.22–1.32	<0.001	1.08	1.04–1.13	<0.001
Rheumatic disease	Presence	0.89	0.82–0.97	0.006			
Peptic ulcer disease	Presence	1.09	1.05–1.14	<0.001	0.96	0.92–1.00	0.049
Mild liver disease	Presence	1.03	0.98–1.08	0.310			
Diabetes without chronic complication	Presence	1.02	0.97–1.09	0.414			
Diabetes with chronic complication	Presence	0.98	0.92–1.05	0.519			
Hemiplegia or paraplegia	Presence	0.52	0.85–1.06	0.332			
Moderate or severe liver disease	Presence	1.97	1.64–2.36	<0.001	2.09	1.74–2.51	<0.001
Metastatic solid tumor	Presence	2.62	2.38–2.89	<0.001	2.39	2.15–2.66	<0.001
Blood loss anemia	Presence	1.22	1.11–1.34	<0.001	1.07	0.97–1.17	0.165
Deficiency anemia	Presence	1.41	1.35–1.47	<0.001	1.18	1.13–1.23	<0.001

Abbreviation: AIDS/HIV = acquired immune deficiency syndrome/human immunodeficiency virus; CI = confidence interval; HR = hazard ratio; NA = not applicable.

^a^ ICD‐10 codes were only considered if they were recorded alone. S72.0 Fracture of femoral neck, S72.1 Pertrochanteric fracture, S72.2 Subtrochanteric fracture.

In the multivariate analysis, we identified the following independent prognostic factors for mortality: male sex, age, fracture site (femoral neck and pertrochanteric), nursing care certification, and several comorbidities (cerebrovascular disease, any malignancy, renal disease, congestive heart failure, chronic pulmonary disease, moderate or severe liver disease, metastatic solid tumor, and deficiency anemia). The results of Cox regression analysis using the test data set are shown in Table S3 and did not differ from those of the training data set.

If we had selected dementia as a potential prognostic factor instead of nursing care certification in the multivariate analysis, the prognostic factors would have remained almost the same, and cerebrovascular disease was newly added despite the fact that its HR was not so extensive (Table S4).

### Use of SHiPS system for mortality after hip fracture

**Fig. 1:**
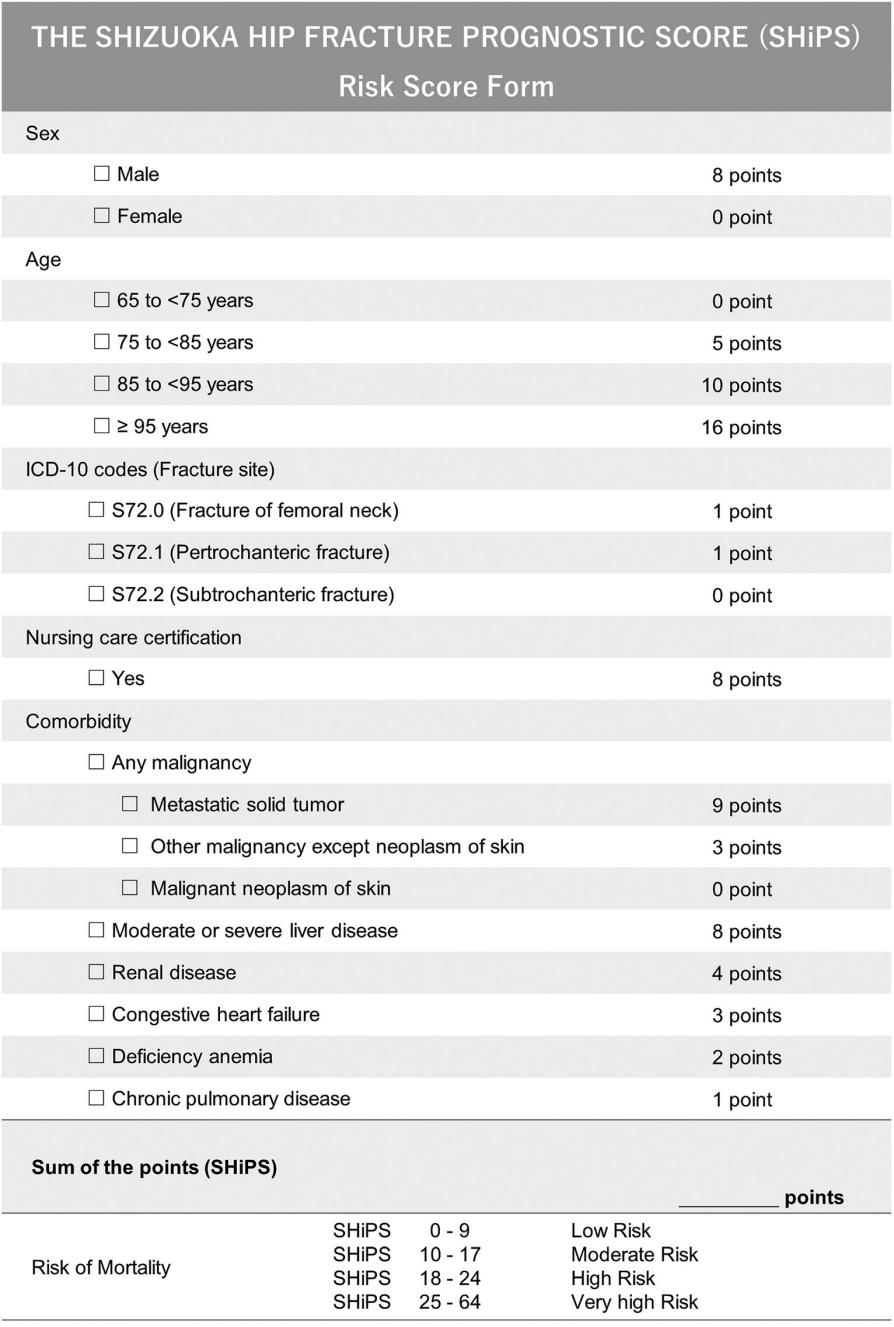
Risk score form for SHiPS. SHiPS = Shizuoka Hip Fracture Prognostic Score.

A scoring system (SHiPS) was constructed to predict post‐fracture mortality by scoring the identified prognostic factors based on their HR resulting from the multivariate Cox regression model. Figure 1 shows the format for calculating risk scores based on the SHiPS. The maximum score was 64 points, with each predictor score ranging from 0 to 16 points. The C‐index (95% CI) was 0.695 (95% CI, 0.691–0.700) in the training data set. By plotting ROC curves using the training data set, the AUC (95% CI) of 1‐year, 3‐year, and 5‐year mortality based on the SHiPS was 0.738 (95% CI, 0.729–0.746), 0.753 (95% CI, 0.746–0.760), and 0.782 (95% CI, 0.772–0.791), respectively.

Figure S2 shows the conditional inference tree fitting for the classification of mortality risk based on the SHiPS, resulting in 0 to 9 points being classified as low risk, 10 to 17 points as moderate risk, 18 to 24 points as high risk, and 25 to 64 points as very high risk (also see Fig. 1). Based on this risk classification, the Kaplan‐Meier curves for the training data set are shown in Fig. S3. The survival probability decreased at all time points in the order of low, moderate, high, and very high‐risk categories.

**Fig. 2:**
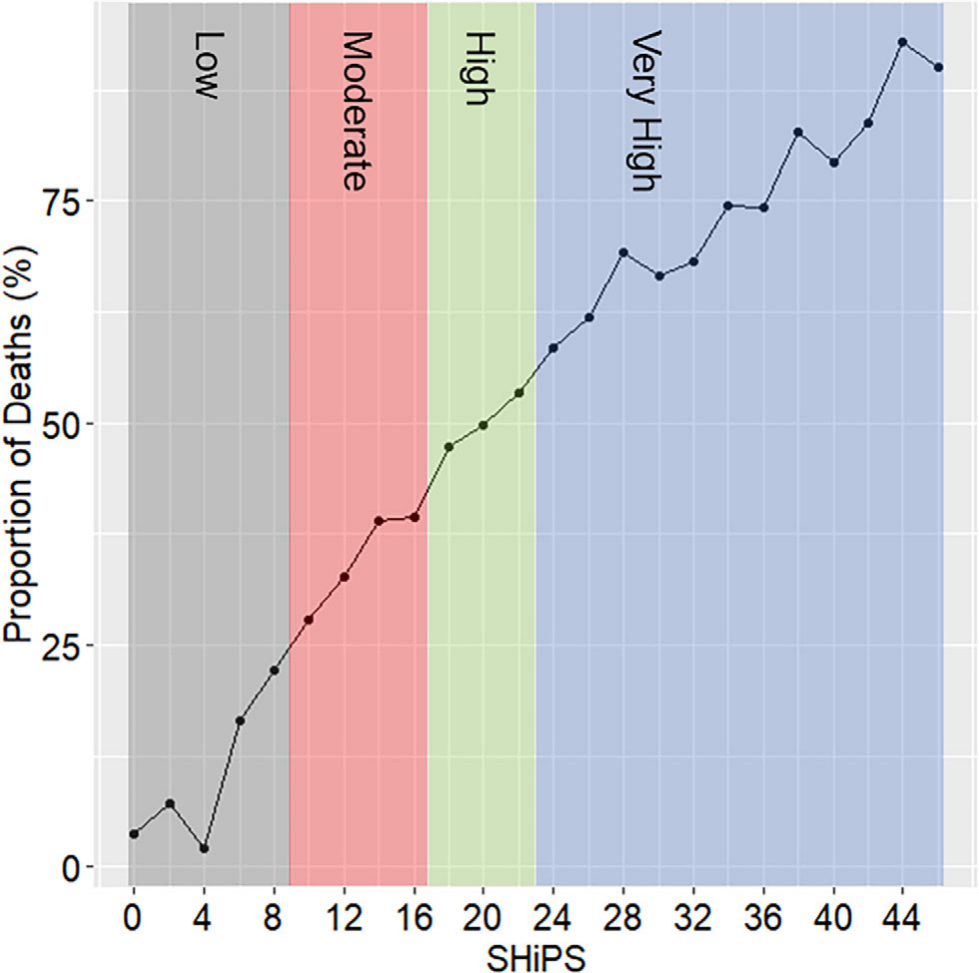
The proportion of deaths according to the SHiPS. The scores of the SHiPS were tallied in 2‐point increments, and the proportion of deaths for each score was calculated. The range of SHiPS for the study population was from 0 to 51. Due to their small number, scores above 46 were grouped together. The mortality risk categories of low (gray), moderate (red), high (green), and very high (blue) were displayed using background colors based on the SHiPS.

The proportion of deaths according to the SHiPS for all patients, including those in the training data set, is shown in Fig. 2. The minimum SHiPS was 0 and the maximum was 51. As the SHiPS increased, the proportion of patients who died also increased.

### Validation of the SHiPS system

The SHiPS system was evaluated for its predictive performance by plotting ROC curves using the test data set (*n* = 14,510). The AUC (95% CI) of 1‐year, 3‐year, and 5‐year mortality based on the SHiPS was 0.718 (95% CI, 0.706–0.729), 0.736 (95% CI, 0.728–0.745), and 0.758 (95% CI, 0.747–0.769), respectively, indicating adequate predictive value for the SHiPS system for as long as 5 years after fracture onset.

**Fig. 3:**
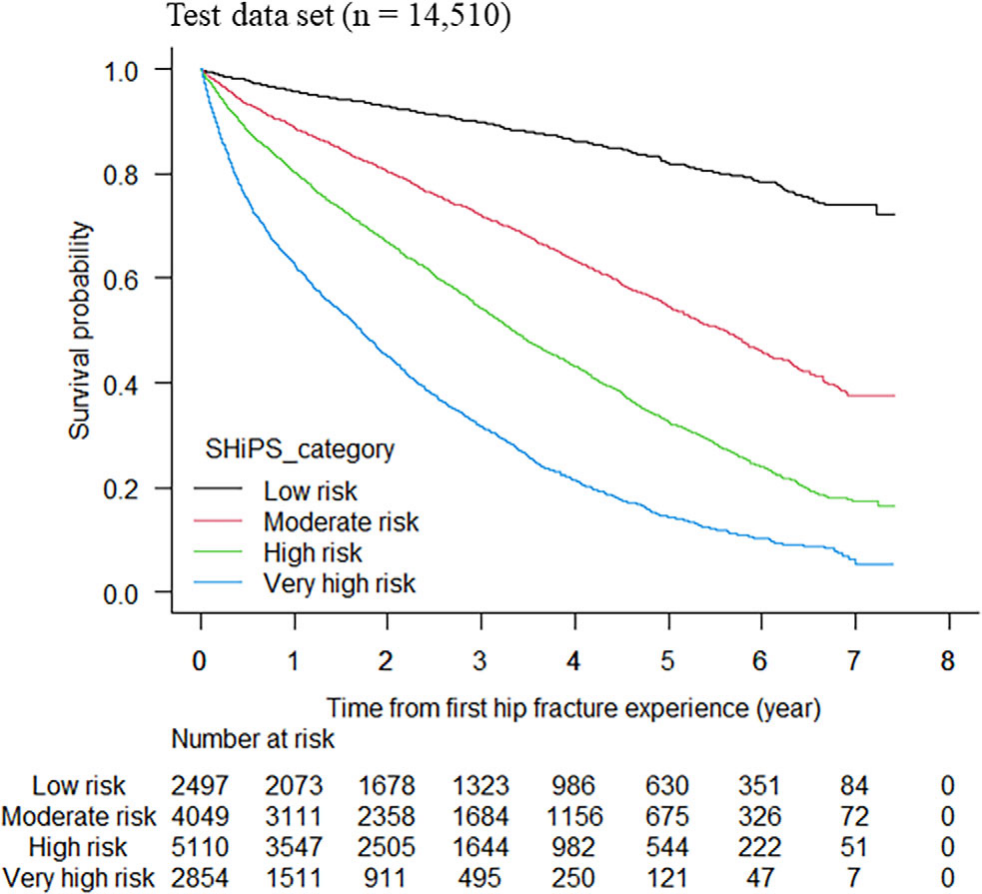
Kaplan‐Meier curves classified by mortality risk category based on SHiPS in test data set.

Figure 3 shows the Kaplan‐Meier curves for the mortality risk category based on the SHiPS in the test data set. Similar to the training data set, the test data set also showed a lower survival probability in the higher risk category throughout the observation period. Additionally, the point estimations of the 1‐year, 3‐year, and 5‐year survival rates are shown in Table 4; worse survival rates were found in the higher risk category at all time points.

**Table 4:** SHiPS Risk Category‐Specific 1‐Year, 3‐Year, and 5‐Year Survival Rates and Their AUCs in Test Data Set

Risk category (SHiPS)	1‐year survival probability (95% CI)			3‐year survival probability (95% CI)			5‐year survival probability (95% CI)		
	Whole test data set	With surgery	Without surgery	Whole test data set	With surgery	Without surgery	Whole test data set	With surgery	Without surgery
Low (0–9)	0.96 (0.95–0.97)	0.97 (0.96–0.97)	0.93 (0.91–0.95)	0.90 (0.90–0.91)	0.91 (0.90–0.93)	0.85 (0.81–0.88)	0.81 (0.79–0.83)	0.83 (0.81–0.85)	0.79 (0.74–0.83)
Moderate (10–17)	0.89 (0.88–0.90)	0.91 (0.90–0.92)	0.83 (0.80–0.85)	0.73 (0.72–0.74)	0.74 (0.72–0.76)	0.65 (0.62–0.68)	0.56 (0.54–0.57)	0.57 (0.54–0.59)	0.49 (0.44–0.53)
High (18–24)	0.80 (0.80–0.82)	0.84 (0.82–0.85)	0.72 (0.69–0.74)	0.54 (0.53–0.55)	0.58 (0.56–0.59)	0.45 (0.42–0.48)	0.31 (0.30–0.33)	0.36 (0.34–0.38)	0.23 (0.20–0.26)
Very high (25–64)	0.63 (0.61–0.64)	0.69 (0.66–0.71)	0.51 (0.48–0.54)	0.29 (0.28–0.31)	0.35 (0.33–0.38)	0.25 (0.22–0.28)	0.13 (0.12–0.14)	0.17 (0.15–0.20)	0.09 (0.07–0.12)
AUC (95% CI)	0.718 (0.701–0.729)	0.711 (0.695–0.727)	0.715 (0.697–0.733)	0.736 (0.728–0.745)	0.729 (0.716–0.743)	0.747 (0.732–0.763)	0.758 (0.747–0.769)	0.742 (0.727–0.757)	0.797 (0.773–0.820)

*Note*: Test data set (*n* = 14,510).

Abbreviation: AUC = area under the receiver operating characteristic curve; CI = confidence interval; SHiPS = Shizuoka Hip Fracture Prognostic Score.

### Predictive performance of SHiPS for patients with or without surgery after hip fracture

The SHiPS was developed to predict the mortality risk based solely on information that can be collected at the time of the fracture, regardless of whether the patient undergoes surgery following fracture onset. We were interested in how well the SHiPS would perform when limited to patients who did or did not undergo surgery. Fig. S4 shows the Kaplan‐Meier curves for the mortality risk category based on the SHiPS for the patients who underwent surgery (*n* = 10,612) (Fig. S4A) and those who did not undergo surgery (*n* = 3,898) (Fig. S4B) in the test data set. The point estimations of the 1‐year, 3‐year, and 5‐year survival rates for each are shown in Table 4. Based on our findings, the SHiPS was considered to work well as an accurate predictor for individual patients with or without surgery in the test data set.

The SHiPS system was also evaluated using ROC curves limited to the patients who did or did not undergo surgery in the test data set (Table 4). Among patients who underwent surgery, the AUC (95% CI) of 1‐year, 3‐year, and 5‐year mortality based on the SHiPS was 0.711 (95% CI, 0.695–0.727), 0.729 (95% CI, 0.716–0.743), and 0.742 (95% CI, 0.727–0.757), respectively. Similarly, that in patients who did not undergo surgery was 0.715 (95% CI, 0.697–0.733), 0.747 (95% CI, 0.732–0.763), and 0.797 (95% CI, 0.773–0.820), respectively. Thus, regardless of whether surgery or a conservative approach was implemented, the predictive value of the SHiPS was adequate for as long as 5 years after fracture onset.

## Discussion

In this study, we analyzed 43,529 advanced‐age patients with hip fracture using a large‐scale population‐based claims database over an 8.5‐year period from 2012 to 2020. We identified preoperative prognostic factors and developed and validated a novel scoring system (the SHiPS) to predict long‐term mortality up to 5 years after hip fracture. The SHiPS consists of information that can be easily obtained before surgery, including sex, age, fracture site, nursing care certification as an indicator of ADL, and several comorbidities (any malignancy, renal disease, congestive heart failure, chronic pulmonary disease, liver disease, metastatic solid tumor, and deficiency anemia). The advantage of the SHiPS is the ability to assess long‐term risk of mortality at the onset of hip fracture, regardless of whether surgery is subsequently performed. Rapid and accurate risk assessment may help anesthesiologists and surgeons to better care for patients from a short‐term perspective and may affect clinical management. In the long term, it may also be helpful in considering treatment strategies alongside rehabilitation and treatment of comorbidities. It also facilitates more effective communication with the patient and his or her relatives to help them understand the prognosis and make rational decisions about clinical management. In the future, it will also help in the development of new medical services and treatment technologies.

The SHiPS is calculated by weighting the HR of each prognostic factor. The total number of points is used to classify patients into low‐risk, moderate‐risk, high‐risk, and very‐high‐risk groups (Fig. 1). For example, an 80‐year‐old man with a femoral neck fracture, nursing care certification, and renal disease would be classified into the very‐high‐risk group with a total SHiPS of 26 points using the risk score form shown in Fig. 1. As shown in Table 4, his predicted 1‐year, 3‐year, and 5‐year survival probability is 62.5%, 29.2%, and 12.8%, respectively.

Our study suggests that the SHiPS can adequately predict postfracture mortality based on preoperative information at the onset time, regardless of whether surgery is performed after the fracture. Surgery was not considered to be a prognostic factor because it was an intermediate variable in our survival analysis and because we aimed to make the SHiPS a useful predictive tool applicable to both surgically and conservatively treated patients. For example, 26.4% of the patients in this study were treated without surgery; this is a significantly higher percentage than the 3.6% in the NHFS development analysis.^(^ ^25^ ^)^ However, from a risk modeling approach^(^ ^43^ ^)^ perspective, our data validating the SHiPS in patients with and without surgery after fracture showed that survival was higher in those with surgery in all risk categories (Table 4, Fig. S4). The association between surgical adaptation and subsequent mortality is an important topic,^(^ ^44^, ^45^ ^)^ and our results showed that patients who underwent surgery had better survival as a consequence. The reason why more people in this study did not undergo surgery is unknown. However, there are two possible reasons why people generally do not opt for surgery. First, surgery cannot be applicable to elderly patients or those with underlying medical conditions for whom the risks associated with surgery and subsequent rehabilitation are too high. Second, patients and their families can choose not to undergo surgery because of concerns about the risks and uncertainties associated with surgery.

Of the predictive factors identified in this study, male sex, older age, and nursing care certification indicating prefracture ADL and dementia have already been reported in many studies,^(^ ^7^, ^8^, ^9^, ^10^, ^11^, ^12^, ^13^, ^14^, ^16^, ^17^, ^18^, ^19^, ^20^, ^21^, ^22^, ^24^, ^25^, ^26^, ^27^ ^)^ and our results are considered reasonable. With respect to the fracture site, intertrochanteric fractures^(^ ^7^ ^)^ and trochanteric fractures^(^ ^9^ ^)^ are reportedly associated with higher mortality than femoral neck fractures. In the present study, however, femoral neck fractures defined as S72.0 in the ICD‐10 and pertrochanteric fractures defined as S72.1 were found to have a slightly more harmful effect on mortality than subtrochanteric fractures defined as S72.2. A low hemoglobin level on admission was identified as a prognostic factor in previous reports,^(^ ^25^, ^27^ ^)^ and our results also showed that deficiency anemia defined by the ECI was a prognostic factor. With respect to comorbidities, six of 17 diagnoses of the CCI were associated with the prognosis in the present study. Some studies, such as the Deyo Charlson index,^(^ ^46^, ^47^ ^)^ weight individual diseases, but unfortunately, most prognostic studies of hip fractures have focused on the number of comorbidities,^(^ ^10^, ^11^, ^17^, ^20^, ^25^ ^)^ making it difficult to understand the involvement of individual diseases. Although some of the conditions identified in this study have been shown to adversely affect mortality (including cerebrovascular disease,^(^ ^12^ ^)^ renal disease,^(^ ^12^, ^16^, ^24^ ^)^ congestive heart failure,^(^ ^13^, ^14^, ^24^ ^)^ chronic lung disease,^(^ ^8^, ^13^, ^24^ ^)^ liver disease,^(^ ^17^, ^21^ ^)^ cancer,^(^ ^12^, ^16^, ^19^, ^24^ ^)^ and anemia^(^ ^23^ ^)^), few studies have comprehensively clarified the extent to which each comorbidity contributes to increased mortality, as in the present study. This is a novel finding. Moreover, if the number of CCI is used as a predictor, it is treated as a single variable. However, if each disease is examined separately, as in this study, the number of predictors increases with the number of diseases, which improves the predictive performance.

Several limitations of this study must be considered. First, the insurance database used in this study does not have records on past hip fracture, smoking habits, BMI, pregnancy, clinical laboratory values, or other unknown factors. Second, due to the significant cost of institutionalization following a fracture, it would be meaningful to use institutionalization‐free survival as an alternative outcome. However, this outcome could not be used in this study due to the difficulty in identifying the codes for institutionalization. Third, the exact surgery date is not recorded in the database; only the month in which the surgery was performed is available. Previous studies have discussed surgical waiting times after hip fracture in the range of hours to days,^(^ ^48^ ^)^ but in our study, we could only analyze the data on a monthly basis. Fourth, the direct cause of death is not recorded in the database. We were also unable to determine whether any rehospitalizations had occurred before death. Fifth, the diagnosis of hip fracture and other comorbidities were based on ICD‐10 codes and were not clinically confirmed, and their validation studies have not been performed. Sixth, this database covered only some residents in Shizuoka prefecture. Still, we minimized the impact of this bias by classifying the cohort by age and performing a multivariate analysis. Seventh, although internal validation was conducted to evaluate the performance of the SHiPS system, the test data set was derived from the same database and may not represent proper external validation. Eighth, nursing care certification is a system unique to Japan and is more widely used than the diagnosis of dementia in Japan. Nursing care certification is an indicator equivalent to disability. Furthermore, we attempted to develop a scoring system for cases in which we adopted the dementia diagnosis as a prognostic factor instead of nursing care certification (Table S4), and we confirmed that the prediction accuracy was sufficient in such cases as well (data not shown). Ninth, this study was conducted in Japan, which has a universal health insurance system. It is expected that similar trends would be observed in countries without universal health insurance systems, although the survival probability would be expected to be slightly lower. Finally, we could not directly compare SHiPS with the previously reported scores because we do not have all the variables that make up previous scores. However, we found it interesting that, by using a completely independent Japanese claims database, the variables such as age, sex, ADL/dementia, anemia, and malignancy were consistent with the previous scores. Despite these limitations, this study allowed us to examine the prognostic factors of post‐fracture mortality and to develop a clinically useful scoring system.

In conclusion, our study demonstrates that the SHiPS is an adequate scoring system for predicting post‐fracture mortality over a long‐term period using preoperative information. Therefore, we believe the SHiPS will be helpful for clinicians, caregivers, and researchers working with the growing number of advanced‐age patients who sustain hip fractures.

## Conflict of Interest Statement

The authors declare no conflicts of interest.

### Peer Review

The peer review history for this article is available at https://www.webofscience.com/api/gateway/wos/peer-review/10.1002/jbm4.10743.

## Supporting information


**Appendix S1.** Supplementary Information

**Fig. S1.** Flow chart illustrating the study population.

**Fig. S2.** The results of conditional inference tree analysis to identify the appropriate mortality risk classification using SHiPS. SHiPS, the Shizuoka Hip Fracture Prognostic Score.

**Fig. S3.** Kaplan‐Meier curves classified by mortality risk category based on SHiPS in training data set.

**Fig. S4.** Kaplan‐Meier curves for groups classified mortality risk category based on SHiPS in patients with or without surgery in test data set. Kaplan‐Meier curves are shown for the patients (A) with and (B) without surgery after fracture onset.
Table S1.
Table S2.
Table S3.
Table S4.

## Data Availability

Based on a data use agreement with regional insurers in Shizuoka Prefecture, we cannot make the analytical data accessible to readers.
