# Supplementary material for: Use of the Shizuoka Hip Fracture Prognostic Score (SHiPS) to Predict Long‐Term Mortality in Patients With Hip Fracture in Japan: A Cohort Study Using the Shizuoka Kokuho Database
Source: JBMR Plus. 2023 Apr 5;7(6):e10743. doi: 10.1002/jbm4.10743 (PMC10241087; doi:10.1002/jbm4.10743)
Supplement: Supplementary file 1 — Appendix S1. Supplementary Information [file JBM4-7-e10743-s003.docx]

**Supplementary Information**

**Supplemental Table 1.** The characteristics of patients with or without surgery after the first onset of hip fracture.

SD, standard deviation; SHiPS, the Shizuoka Hip Fracture Prognostic Score. *ICD-10 codes are only considered if they are recorded alone. S72.0 Fracture of femoral neck, S72.1 Pertrochanteric fracture, S72.2 Subtrochanteric fracture.

**Supplemental Table 2.** Spearman’s rank correlation coefficients for predictors in the training dataset.

Bold type indicates a correlation of > 0.4.

**Supplemental Table 3.** The results of univariate and multivariate Cox regression analysis for predictive factors of post-fracture mortality adopting nursing care certification as one of the prognostic factors in the test dataset.

CI, confidence interval; HR, hazard ratio; NA, not applicable; *ICD-10 codes are only considered if they are recorded alone. S72.0 Fracture of femoral neck, S72.1 Pertrochanteric fracture, S72.2 Subtrochanteric fracture.

**Supplemental Table 4.** The results of univariate and multivariate Cox regression analysis for predictive factors of post-fracture mortality adopting dementia in the training dataset.

CI, confidence interval; HR, hazard ratio; NA, not applicable; *ICD-10 codes are only considered if they are recorded alone. S72.0 Fracture of femoral neck, S72.1 Pertrochanteric fracture, S72.2 Subtrochanteric fracture. **Score for adopting dementia.

**Supplemental Figure 1.** Flow chart illustrating the study population.

**Supplemental Figure 2.** The results of conditional inference tree analysis to identify the appropriate mortality risk classification using SHiPS.

SHiPS, the Shizuoka Hip Fracture Prognostic Score.

**Supplemental Figure 3.** Kaplan–Meier curves classified by mortality risk category based on SHiPS in training data set.

**Supplemental Figure 4.** Kaplan–Meier curves for groups classified mortality risk category based on SHiPS in patients with or without surgery in test data set.

Kaplan–Meier curves are shown for the patients (A) with and (B) without surgery after fracture onset.

**Supplemental table 1.** The characteristics of patients with or without surgery after the first onset of hip fracture.

| **Variable** | **Category** | **Patients with surgery (n = 32,025)** |  | **Patients without surgery (n = 11,504)** | ***P*-value** |
| --- | --- | --- | --- | --- | --- |
|  |  | **Number (%)** |  | **Number (%)** |  |
| **SHiPS** | Mean ± SD | 11.4 ± 5.1 |  | 12.6 ± 5.3 | < 0.001 |
| **Sex** | Male | 6,417 (20.0) |  | 2,613 (22.7) | < 0.001 |
|  | Female | 25,608 (80.0) |  | 8,891 (77.3) |  |
| **Age** | Mean ± SD | 85.0 ± 7.1 |  | 85.5 ± 7.8 |  |
|  | 65 to <75 years | 2,724 (8.5) |  | 1,130 (9.8) | < 0.001 |
|  | 75 to <85 years | 11,242 (35.1) |  | 3,619 (31.5) |  |
|  | 85 to <95 years | 15,610 (48.7) |  | 5,452 (47.4) |  |
|  | ≥95 years | 2,449 (7.6) |  | 1,303 (11.3) |  |
| **Season of onset** | Jan - Mar | 8,676 (27.1) |  | 2,978(25.9) | < 0.001 |
|  | Apr - Jun | 7,480 (23.4) |  | 2,866 (24.9) |  |
|  | Jul - Sep | 7,374 (23.0) |  | 2,762 (24.0) |  |
|  | Oct - Dec | 8,495 (26.5) |  | 2,898 (25.2) |  |
| **Fracture site*** |  |  |  |  |  |
| S72.0 | Presence | 14,227 (44.4) |  | 7,685 (66.8) | < 0.001 |
| S72.1 | Presence | 11,987 (37.4) |  | 3,057 (26.6) | < 0.001 |
| S72.2 | Presence | 390 (1.2) |  | 75 (0.7) | < 0.001 |
| **Nursing care certification** | Presence | 11,754 (54.9) |  | 4,894 (64.3) | < 0.001 |
| **Comorbidity** |  |  |  |  |  |
| Cerebrovascular disease | Presence | 10,851 (33.9) |  | 4,164 (36.2) | < 0.001 |
| Any malignancy | Presence | 4,023 (12.6) |  | 1,671 (14.5) | < 0.001 |
| Dementia | Presence | 10,436 (32.6) |  | 3,961 (34.4) | < 0.001 |
| AIDS/HIV | Presence | 9 (0.0) |  | 4 (0.0) | 0.755 |
| Myocardial infarction | Presence | 1,239 (3.9) |  | 617 (5.4) | < 0.001 |
| Renal disease | Presence | 3,077 (9.6) |  | 1,394 (12.1) | < 0.001 |
| Congestive heart failure | Presence | 10,876 (34.0) |  | 4,587 (39.9) | < 0.001 |
| Peripheral vascular disease | Presence | 4,799 (15.0) |  | 1,819 (15.8) | 0.035 |
| Chronic pulmonary disease | Presence | 7,855 (24.5) |  | 3,138 (27.3) | < 0.001 |
| Rheumatic disease | Presence | 1,609 (5.0) |  | 622 (5.4) | 0.115 |
| Peptic ulcer disease | Presence | 7,740 (24.2) |  | 2,967 (25.8) | 0.001 |
| Mild liver disease | Presence | 5,112 (16.0) |  | 1,756 (15.3) | 0.079 |
| Diabetes without chronic complication | Presence | 3,615 (11.3) |  | 891 (7.7) | < 0.001 |
| Diabetes with chronic complication | Presence | 2,467 (7.7) |  | 821 (7.1) | 0.048 |
| Hemiplegia or paraplegia | Presence | 836 (2.6) |  | 354 (3.1) | 0.009 |
| Moderate or severe liver disease | Presence | 183 (0.6) |  | 104 (0.9) | < 0.001 |
| Metastatic solid tumor | Presence | 620 (1.9) |  | 335 (2.9) | < 0.001 |
| Blood loss anemia | Presence | 1,347 (4.2) |  | 167 (1.5) | < 0.001 |
| Deficiency anemia | Presence | 7,349 (22.9) |  | 2,539 (22.1) | 0.055 |

SD, standard deviation; SHiPS, the Shizuoka Hip Fracture Prognostic Score. *ICD-10 codes are only considered if they are recorded alone. S72.0 Fracture of femoral neck, S72.1 Pertrochanteric fracture, S72.2 Subtrochanteric fracture.

**Supplemental table 2.** Spearman’s rank correlation coefficients for predictors in the training dataset.

|  | Sex | Age | Fracture sites S72.0 | Fracture sites S72.1 | Nursing care certification | Cerebro-vascular disease | Any malig-nancy | Dementia | Myo-cardial infarction | Renal disease | Congestive heart failure | Peripheral vascular disease | Chronic pulmonary disease | Peptic ulcer disease | Moderate or severe liver disease | Metastatic solid tumor | Blood loss anemia | Deficien-cy anemia |
| --- | --- | --- | --- | --- | --- | --- | --- | --- | --- | --- | --- | --- | --- | --- | --- | --- | --- | --- |
| Sex | 1 |  |  |  |  |  |  |  |  |  |  |  |  |  |  |  |  |  |
| Age | -0.12 | 1 |  |  |  |  |  |  |  |  |  |  |  |  |  |  |  |  |
| Fracture sites S72.0 | 0.03 | -0.17 | 1 |  |  |  |  |  |  |  |  |  |  |  |  |  |  |  |
| Fracture sites S72.1 | 0 | 0.15 | -0.73 | 1 |  |  |  |  |  |  |  |  |  |  |  |  |  |  |
| Nursing care certification | -0.05 | 0.32 | -0.04 | 0.04 | 1 |  |  |  |  |  |  |  |  |  |  |  |  |  |
| Cerebro-  vascular disease | 0.07 | 0.01 | 0.01 | 0 | 0.15 | 1 |  |  |  |  |  |  |  |  |  |  |  |  |
| Any malignancy | 0.18 | 0.03 | 0.02 | -0.01 | -0.06 | 0 | 1 |  |  |  |  |  |  |  |  |  |  |  |
| Dementia | -0.05 | 0.17 | -0.02 | 0.01 | **0.43** | 0.1 | -0.05 | 1 |  |  |  |  |  |  |  |  |  |  |
| Myocardial infarction | 0.08 | 0.01 | 0 | 0 | 0.01 | 0.06 | 0.03 | -0.01 | 1 |  |  |  |  |  |  |  |  |  |
| Renal disease | 0.10 | 0.01 | 0 | 0.01 | 0 | 0.04 | 0.04 | -0.02 | 0.1 | 1 |  |  |  |  |  |  |  |  |
| Congestive heart failure | 0.02 | 0.17 | -0.03 | 0.03 | 0.13 | 0.12 | 0.03 | 0.06 | 0.18 | 0.21 | 1 |  |  |  |  |  |  |  |
| Peripheral vascular disease | 0.06 | -0.02 | 0.01 | -0.01 | -0.03 | 0.11 | 0.05 | -0.05 | 0.09 | 0.13 | 0.13 | 1 |  |  |  |  |  |  |
| Chronic pulmonary disease | 0.07 | 0.03 | 0 | -0.01 | 0.03 | 0.04 | 0.06 | -0.01 | 0.04 | 0.05 | 0.12 | 0.14 | 1 |  |  |  |  |  |
| Peptic ulcer disease | 0.05 | -0.06 | 0.02 | -0.02 | -0.02 | 0.1 | 0.12 | -0.04 | 0.06 | 0.09 | 0.12 | 0.12 | 0.1 | 1 |  |  |  |  |
| Moderate or severe liver disease | 0.02 | -0.05 | 0 | 0 | -0.02 | 0 | 0.06 | -0.01 | -0.01 | 0.03 | 0.02 | 0 | 0.01 | 0.05 | 1 |  |  |  |
| Metastatic solid tumor | 0 | -0.07 | 0.01 | -0.01 | -0.04 | -0.01 | 0.34 | -0.04 | 0.01 | 0.01 | -0.01 | 0.01 | 0.02 | 0.05 | 0.05 | 1 |  |  |
| Blood loss anemia | 0 | 0.03 | -0.06 | 0.05 | 0.02 | 0.02 | 0.02 | 0.01 | 0.22 | 0.03 | 0.02 | 0.01 | 0.01 | 0.02 | 0.02 | 0 | 1 |  |
| Deficiency anemia | -0.03 | 0.03 | -0.05 | 0.04 | 0.03 | 0.04 | 0.11 | -0.01 | 0.06 | 0.23 | 0.17 | 0.11 | 0.06 | 0.15 | 0.04 | 0.04 | 0.03 | 1 |

Bold type indicates a correlation of > 0.4.

**Supplemental Table 3.** The results of univariate and multivariate Cox regression analysis for predictive factors of post-fracture mortality adopting nursing care certification as one of the prognostic factors in the test dataset.

| **Variable (reference)** | **Category** | **Test dataset (n = 14,510)** | | | | | | |
| --- | --- | --- | --- | --- | --- | --- | --- | --- |
|  |  | **Univariable model** | | |  | **Multivariable model** | | |
|  |  | **HR** | **95% CI** | ***P*-value** |  | **HR** | **95% CI** | ***P* -value** |
| **Sex** (Female) | Male | 1.89 | 1.79-2.00 | < 0.001 |  | 2.08 | 1.97-2.21 | < 0.001 |
| **Age** (65 to <75 years) | 75 to <85 years | 1.79 | 1.57-2.05 | < 0.001 |  | 1.57 | 1.37-1.80 | < 0.001 |
|  | 85 to <95 years | 3.29 | 2.89-3.75 | < 0.001 |  | 2.67 | 2.34-3.05 | < 0.001 |
|  | ≥95 years | 5.24 | 4.53-6.05 | < 0.001 |  | 4.29 | 3.70-4.98 | < 0.001 |
| **Season of onset** (Jan - Mar) | Apr - Jun | 0.94 | 0.88-1.01 | 0.093 |  |  |  |  |
|  | Jul - Sep | 0.94 | 0.94-1.01 | 0.080 |  |  |  |  |
|  | Oct - Dec | 0.99 | 0.99-1.06 | 0.777 |  |  |  |  |
| **Fracture site*** (absence) |  |  |  |  |  |  |  |  |
| S72.0 | Presence | 0.90 | 0.86-0.95 | < 0.001 |  | 1.19 | 1.10-1.28 | < 0.001 |
| S72.1 | Presence | 1.21 | 1.15-1.28 | < 0.001 |  | 1.23 | 1.14-1.33 | < 0.001 |
| S72.2 | Presence | 0.89 | 0.68-1.16 | 0.383 |  |  |  |  |
| **Nursing care certification** (absence) | Presence | 2.41 | 2.23-2.55 | < 0.001 |  | 2.09 | 1.98-2.22 | < 0.001 |
| **Comorbidity** (absence) |  |  |  |  |  |  |  |  |
| Cerebrovascular disease | Presence | 1.18 | 1.12-1.24 | < 0.001 |  | 0.97 | 0.92-1.02 | 0.250 |
| Any malignancy | Presence | 1.64 | 1.54-1.76 | < 0.001 |  | 1.36 | 1.26-1.47 | < 0.001 |
| Dementia | Presence | 1.68 | 1.60-1.77 | < 0.001 |  | NA | NA | NA |
| AIDS/HIV | Presence | 1.01 | 0.32-3.12 | 0.991 |  |  |  |  |
| Myocardial infarction | Presence | 1.56 | 1.40-1.74 | < 0.001 |  | 1.20 | 1.08-1.34 | 0.001 |
| Renal disease | Presence | 1.73 | 1.61-1.87 | < 0.001 |  | 1.38 | 1.28-1.50 | < 0.001 |
| Congestive heart failure | Presence | 1.62 | 1.54-1.70 | < 0.001 |  | 1.23 | 1.16-1.29 | < 0.001 |
| Peripheral vascular disease | Presence | 1.08 | 1.01-1.15 | 0.032 |  |  |  |  |
| Chronic pulmonary disease | Presence | 1.27 | 1.20-1.34 | < 0.001 |  | 1.12 | 1.06-1.18 | < 0.001 |
| Rheumatic disease | Presence | 0.99 | 0.88-1.11 | 0.812 |  |  |  |  |
| Peptic ulcer disease | Presence | 1.11 | 1.05-1.17 | < 0.001 |  | 0.97 | 0.92-1.03 | 0.366 |
| Mild liver disease | Presence | 0.96 | 0.95-1.12 | 0.198 |  |  |  |  |
| Diabetes without chronic complication | Presence | 0.94 | 0.86-1.04 | 0.241 |  |  |  |  |
| Diabetes with chronic complication | Presence | 0.98 | 0.92-1.05 | 0.519 |  |  |  |  |
| Hemiplegia or paraplegia | Presence | 0.99 | 0.85-1.14 | 0.863 |  |  |  |  |
| Moderate or severe liver disease | Presence | 2.66 | 2.11-3.34 | < 0.001 |  | 2.19 | 1.73-2.76 | < 0.001 |
| Metastatic solid tumor | Presence | 2.71 | 2.37-3.09 | < 0.001 |  | 2.20 | 1.90-2.54 | < 0.001 |
| Blood loss anemia | Presence | 1.24 | 1.09-1.42 | 0.001 |  |  |  |  |
| Deficiency anemia | Presence | 1.41 | 1.33-1.49 | < 0.001 |  | 1.16 | 1.09-1.23 | < 0.001 |

CI, confidence interval; HR, hazard ratio; NA, not applicable; *ICD-10 codes are only considered if they are recorded alone. S72.0 Fracture of femoral neck, S72.1 Pertrochanteric fracture, S72.2 Subtrochanteric fracture.

**Supplemental Table 4.** The results of multivariate Cox regression analysis for predictive factors of post-fracture mortality adopting dementia in the training dataset.

| **Variable (reference)** | **Category** | **Training dataset (n = 29,019)** | | | |
| --- | --- | --- | --- | --- | --- |
|  |  | **Multivariable model** | | | **Score**** |
|  |  | **HR** | **95% CI** | ***P*-value** |  |
| **Sex** (Female) | Male | 2.01 | 1.92-2.09 | < 0.001 | 7 |
| **Age** (65 to <75 years) | 75 to <85 years | 1.63 | 1.49-1.79 | < 0.001 | 5 |
|  | 85 to <95 years | 3.02 | 2.76-3.30 | < 0.001 | 11 |
|  | ≥95 years | 5.75 | 5.20-6.37 | < 0.001 | 17 |
| **Season of onset** (Jan - Mar) | Apr - Jun |  |  |  |  |
|  | Jul - Sep |  |  |  |  |
|  | Oct - Dec |  |  |  |  |
| **Fracture site*** (absence) |  |  |  |  |  |
| S72.0 | Presence | 1.12 | 1.06-1.18 | < 0.001 | 1 |
| S72.1 | Presence | 1.15 | 1.09-1.21 | < 0.001 | 1 |
| S72.2 | Presence |  |  |  |  |
| **Nursing care certification** (absence) | Presence | NA | NA | NA | NA |
| **Comorbidity** (absence) |  |  |  |  |  |
| Cerebrovascular disease | Presence | 1.10 | 1.06-1.14 | < 0.001 | 1 |
| Any malignancy | Presence | 1.28 | 1.21-1.35 | < 0.001 | 2 |
| Dementia | Presence | 1.55 | 1.49-1.61 | < 0.001 | 4 |
| AIDS/HIV | Presence |  |  |  |  |
| Myocardial infarction | Presence | 1.12 | 1.04-1.22 | 0.004 |  |
| Renal disease | Presence | 1.44 | 1.36-1.52 | < 0.001 | 4 |
| Congestive heart failure | Presence | 1.37 | 1.31-1.42 | < 0.001 | 3 |
| Peripheral vascular disease | Presence | 0.97 | 0.92-1.02 | 0.205 |  |
| Chronic pulmonary disease | Presence | 1.11 | 1.07-1.15 | < 0.001 | 1 |
| Rheumatic disease | Presence |  |  |  |  |
| Peptic ulcer disease | Presence | 0.97 | 0.93-1.01 | 0.146 |  |
| Mild liver disease | Presence |  |  |  |  |
| Diabetes without chronic complication | Presence |  |  |  |  |
| Diabetes with chronic complication | Presence |  |  |  |  |
| Hemiplegia or paraplegia | Presence |  |  |  |  |
| Moderate or severe liver disease | Presence | 2.14 | 1.78-2.57 | < 0.001 | 8 |
| Metastatic solid tumor | Presence | 2.34 | 2.11-2.61 | < 0.001 | 9 |
| Blood loss anemia | Presence |  |  |  |  |
| Deficiency anemia | Presence | 1.18 | 1.13-1.23 | < 0.001 | 2 |

CI, confidence interval; HR, hazard ratio; NA, not applicable; *ICD-10 codes are only considered if they are recorded alone. S72.0 Fracture of femoral neck, S72.1 Pertrochanteric fracture, S72.2 Subtrochanteric fracture. **Score for adopting dementia.
