# Supplementary figures and images for: Use of the Shizuoka Hip Fracture Prognostic Score (SHiPS) to Predict Long‐Term Mortality in Patients With Hip Fracture in Japan: A Cohort Study Using the Shizuoka Kokuho Database
Source: JBMR Plus. 2023 Apr 5;7(6):e10743. doi: 10.1002/jbm4.10743 (PMC10241087; doi:10.1002/jbm4.10743)

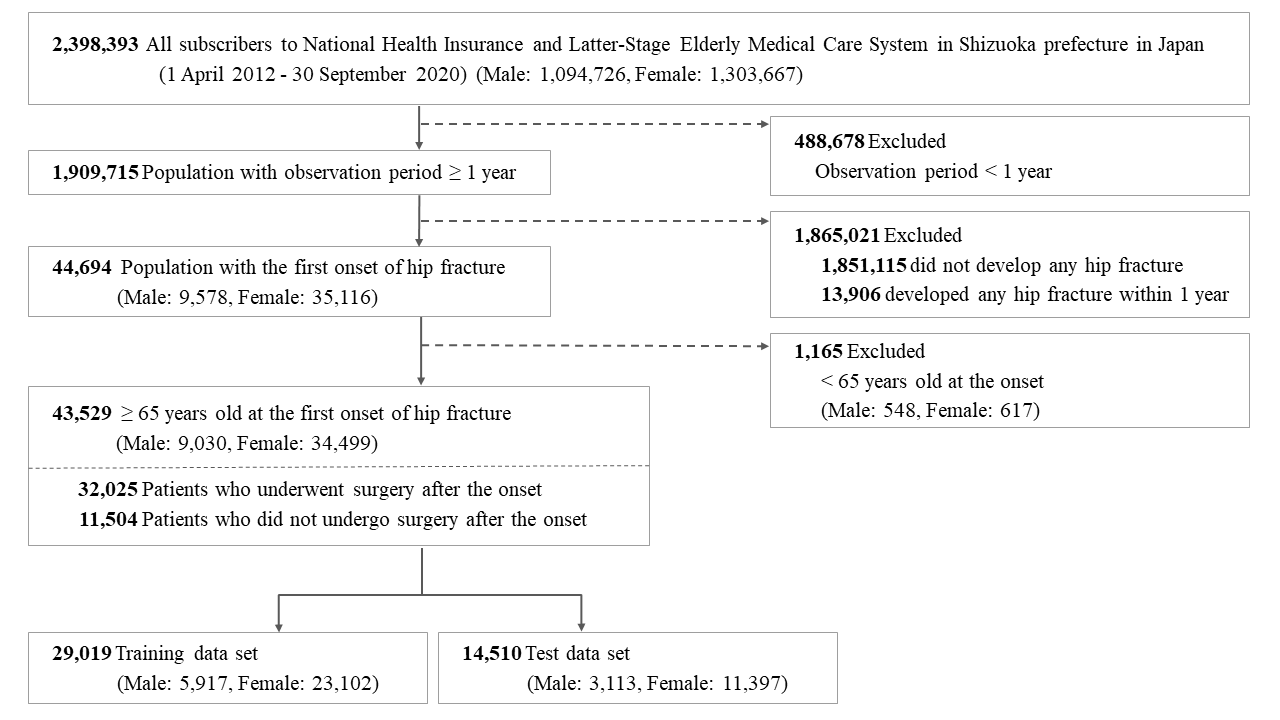

Supplement: Supplementary file 2 — Fig. S1. Flow chart illustrating the study population. [file JBM4-7-e10743-s001.tif]

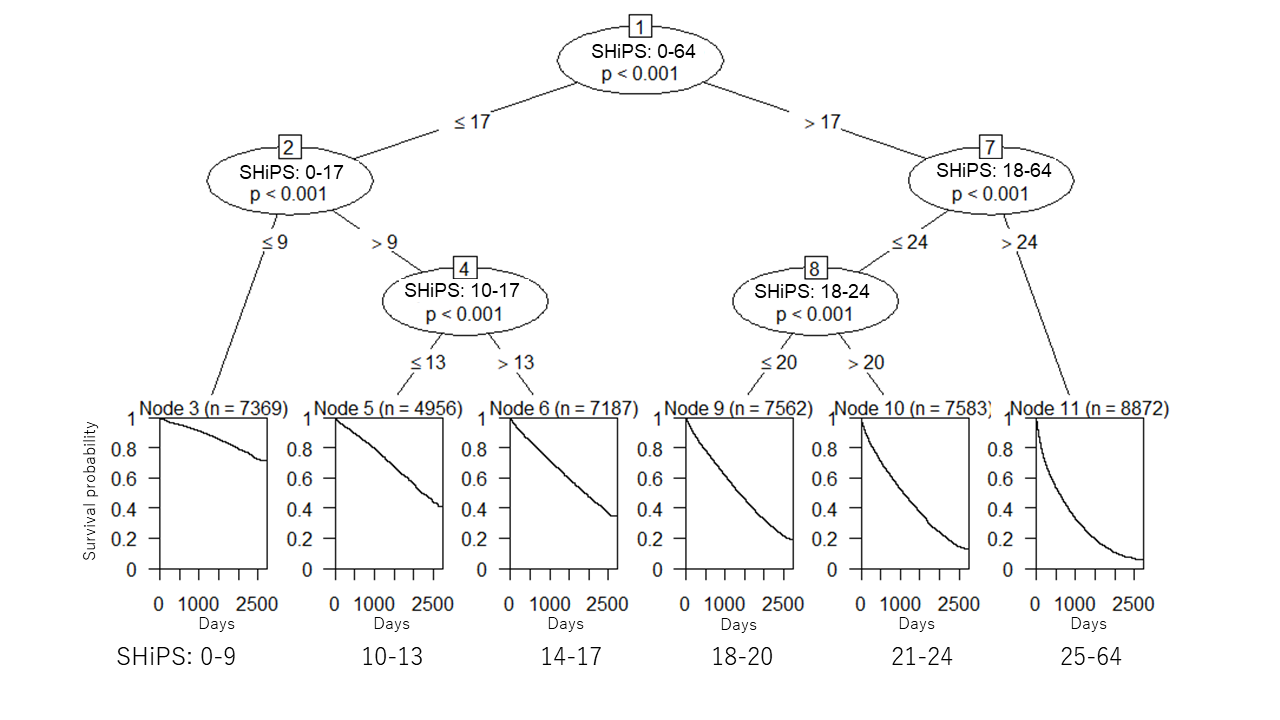

Supplement: Supplementary file 3 — Fig. S2. The results of conditional inference tree analysis to identify the appropriate mortality risk classification using SHiPS. SHiPS, the Shizuoka Hip Fracture Prognostic Score. [file JBM4-7-e10743-s004.tif]

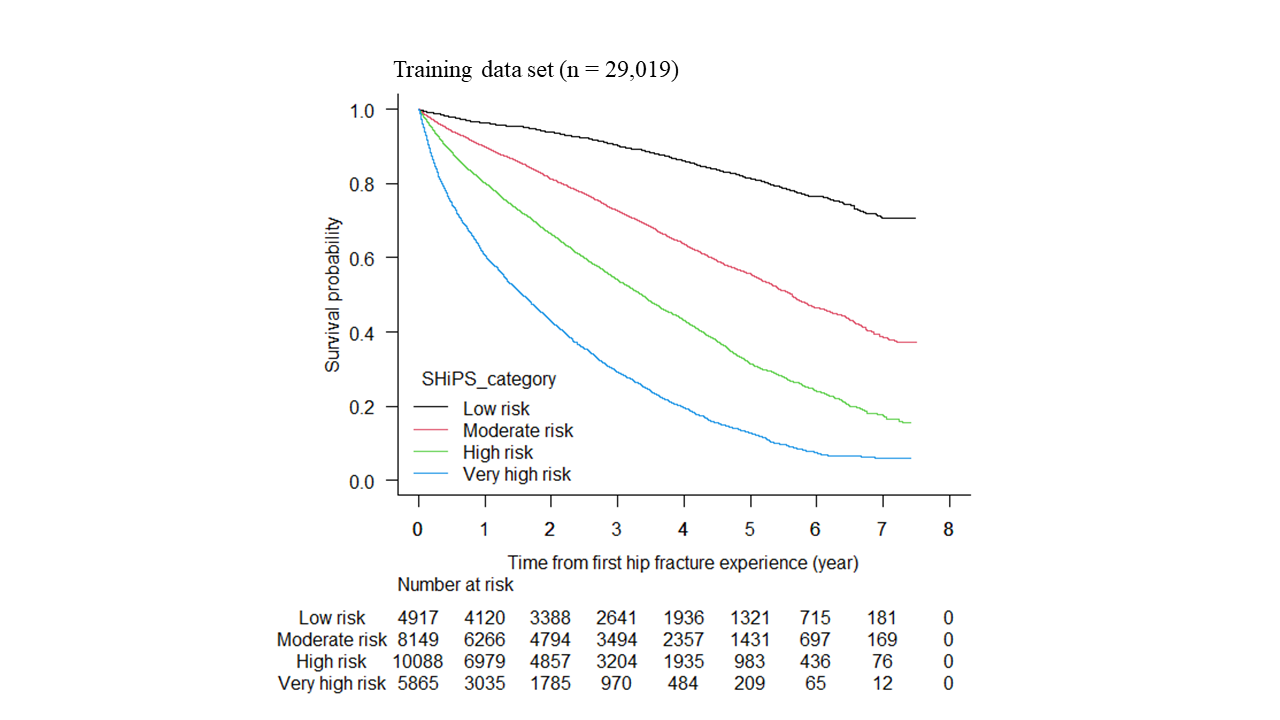

Supplement: Supplementary file 4 — Fig. S3. Kaplan‐Meier curves classified by mortality risk category based on SHiPS in training data set. [file JBM4-7-e10743-s005.tif]

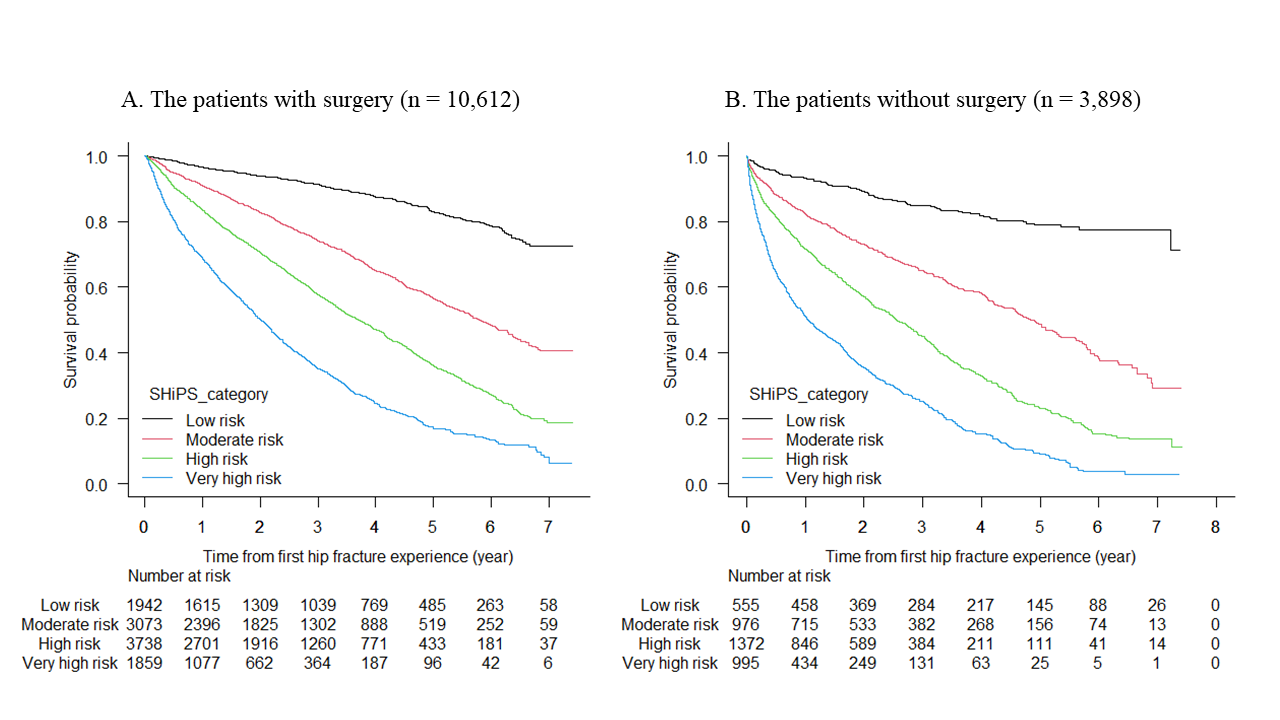

Supplement: Supplementary file 5 — Fig. S4. Kaplan‐Meier curves for groups classified mortality risk category based on SHiPS in patients with or without surgery in test data set. Kaplan‐Meier curves are shown for the patients (A) with and (B) without surgery after fracture onset. Table S1. Table S2. Table S3. Table S4. [file JBM4-7-e10743-s002.tif]
